# Characteristics Associated With Financial or Non-financial Barriers to Healthcare in a Universal Health Insurance System: A Longitudinal Analysis of Korea Health Panel Survey Data

**DOI:** 10.3389/fpubh.2022.828318

**Published:** 2022-03-08

**Authors:** Woojin Chung

**Affiliations:** ^1^Department of Health Policy and Management, Graduate School of Public Health, Yonsei University, Seoul, South Korea; ^2^Institute of Health Services Research, Yonsei University, Seoul, South Korea

**Keywords:** unmet healthcare needs, financial and non-financial healthcare barriers, panel multinomial logistic regression, average adjusted probability, Korea Health Panel survey, South Korea

## Abstract

While many studies have explored the financial barriers to healthcare, there is little evidence regarding the non-financial barriers to healthcare. This study identified characteristics associated with financial and non-financial barriers to healthcare and quantified the effects of these characteristics in South Korea, using a nationally representative longitudinal survey dataset. Overall, 68,930 observations of 16,535 individuals aged 19 years and above were sampled from Korea Health Panel survey data (2014–2018). From self-reported information about respondents' experiences of unmet healthcare needs, a trichotomous dependent variable—no barrier, non-financial barrier, and financial barrier—was derived. Sociodemographics, physical and health conditions were included as explanatory variables. The average adjusted probability (AAP) of experiencing each barrier was predicted using multivariable and panel multinomial logistic regression analyses. According to the results, the percentage of people experiencing non-financial barriers was much higher than that of people experiencing financial barriers in 2018 (9.6 vs. 2.5%). Women showed higher AAPs of experiencing both non-financial (9.9 vs. 8.3%) and financial barriers (3.6 vs. 2.5%) than men. Men living in the Seoul metropolitan area showed higher AAPs of experiencing non-financial (8.7 vs. 8.0%) and financial barriers (3.4 vs. 2.1%) than those living outside it. Household income showed no significant associations in the AAP of experiencing a non-financial barrier. People with a functional limitation exhibited a higher AAP of experiencing a non-financial barrier, for both men (17.8 vs. 7.8%) and women (17.4 vs. 9.0%), than those without it. In conclusion, people in South Korea, like those in most European countries, fail to meet their healthcare needs more often due to non-financial barriers than financial barriers. In addition, the characteristics associated with non-financial barriers to healthcare differed from those associated with financial barriers. This finding suggests that although financial barriers may be minimised through various policies, a considerable degree of unmet healthcare needs and disparity among individuals is very likely to persist due to non-financial barriers. Therefore, current universal health insurance systems need targeted policy instruments to minimise non-financial barriers to healthcare to ensure effective universal health coverage.

## Introduction

To ensure that citizens have timely and adequate access to healthcare services, many countries strive to identify and minimise barriers to healthcare by providing a universal health coverage system (1, 2). Furthermore, efforts to lower financial barriers to healthcare, irrespective of individuals' income, such as reducing the financial burden on individuals or households in obtaining healthcare services, have often been undertaken. Numerous studies have emphasised the detrimental effects of financial barriers to healthcare on the utilisation of healthcare services (3, 4). An awareness of such barriers and a determination that healthcare coverage not be compromised are clearly expressed in various definitions of universal health coverage: “Universal health coverage means that all people have access to the health services they need, when and where they need them, *without financial hardship*” (1) and “Universal health coverage is about ensuring that people have access to the healthcare they need *without suffering financial hardship*” (2).

However, in the last decade, certain studies have warned against overlooking the importance of non-financial barriers to healthcare (5–8). Non-financial barriers continue to be a serious public health threat to disadvantaged populations in many countries. A recent report documented that, in 2019, 2.2% of those aged 16 years and above in European countries did not receive healthcare due to a non-financial barrier in the 12 months prior to taking the survey (9). This percentage is much higher than that of those who faced a financial barrier (0.9%).

Nevertheless, no study has distinguished between non-financial and financial barriers or sought to determine which barrier type negatively affects individuals' access to healthcare more severely. Several researchers have categorised barriers to healthcare into different groups (10–13). For example, the cost of utilising healthcare services, that is, the financial barrier to healthcare is categorised in terms of “affordability” in one study (11) and in terms of “accessibility” in another (10). As these categorisations vary largely across studies in terms of number and content based on their study purposes, it is difficult to examine the association between individuals' characteristics and their experience of non-financial and financial barriers. Consequently, it can be challenging to examine the association between individuals' characteristics and their experience of non-financial and financial barriers, which further impedes the development of appropriate policies. Further, no study has performed an in-depth analysis regarding how individual characteristics associated with non-financial barriers differ from those associated with financial barriers in a country with a universal health insurance system.

Therefore, the present study aimed to address this substantial gap in the literature by categorising barriers leading to unmet healthcare needs into two types, namely, non-financial and financial barriers, and to determine how these two types of barrier affected healthcare, through analysing self-reported data on unmet healthcare needs. A nationwide panel survey dataset from South Korea was used to conduct multivariable, panel multinomial logit model analyses and explore the characteristics associated with each type of barrier to healthcare. It is important to examine the factors associated with both non-financial and financial barriers to aid researchers in developing and testing new theories about the utilisation of healthcare services. Furthermore, the study results can help policy-makers apply targeted policies to reduce each type of barrier to healthcare effectively. Given that advanced countries/regions in Asia such as Japan, Chinese Taipei, and South Korea have universal health insurance systems based on those of European countries and modified to fit into their own socioeconomic circumstances, the results of the present study are likely to provide insights to countries/regions in both Europe and Asia regarding potential improvements to their universal health coverage systems.

## South Korea's Healthcare System

South Korea (hereafter, Korea) has provided financial support for healthcare to its entire population *via* two public financially secured healthcare protection programmes since 1989, namely, the Medical Care Aid (MCA) programme, a public in-kind aid programme for the poor, which covers ~3% of the population, and the National Health Insurance (NHI) programme, a social health insurance programme for the remaining population (14). The NHI is operated by a single public funder, the National Health Insurance Service (NHIS), under the direction and supervision of the Korean Ministry of Health and Welfare. As such, both the contribution schedule and benefits coverage are identical throughout the country. The financing of the NHI depends mainly on contributions imposed on employment income and property. Healthcare delivery relies very heavily on private providers, and physicians and hospitals—whether public or private—are mostly reimbursed based on fee-for-service payment.

Individuals can select the physicians and hospitals of their choice for their outpatient needs. Most clinics in Korea also provide patients with inpatient services, which may be because the referral system is not well-established. Another reason for this may be the lack of primary care physicians such as general practitioners in the United Kingdom (UK) and gatekeepers in managed care organisations in the United States of America (USA). In Korea, the percentage of general practitioners is extremely low, with 6% recorded in 2017 (15). Non-essential healthcare services not covered under the NHI programme are provided along with essential healthcare services and the prices for such services are not regulated by the government. Therefore, the out-of-pocket payments for non-essential healthcare services are incurred through co-payments (or coinsurance rates) and expenses for healthcare services not covered by the NHI programmes. Individuals pay out-of-pocket payments either through direct payment or private health insurance, or both (14).

Compared to the Organisation for Economic Cooperation and Development (OECD) member countries, the number of practising doctors per 1,000 people in Korea is very low (2.3 in 2017), as is the number of practising nurses per 1,000 people (6.9 in 2017) (15). Although the annual growth rate in health expenditure per capita between 2013 and 2018 was high (7.3%), health expenditure as a percentage of the gross domestic product was lower (8.1% in 2018) than the average percentage in 36 OECD member countries (8.8%). The rate of healthcare utilisation was found to be high, with 16.6 doctor consultations reported per person, and the average length of stay in hospital was 18.5 days, in 2017. Life expectancy at birth was higher (82.7 years in 2017) than that in 36 OECD member countries (80.7 years in 2017), but the percentage of the population aged 65 years and above was lower (13.8% in 2017) than that in 36 OECD member countries (17.4% in 2017) (15).

## Methods

### Data Source and Study Sample

This study analysed data collected from the Korea Health Panel (KHP) survey (version 1.7). The KHP survey is a national non-institutionalised civilian population survey conducted by the Korea Institute for Health and Social Affairs and the NHIS. Sample households are selected using two-stage, clustered probability sampling on population census data collated by Statistics Korea. The KHP survey includes data from all eligible household members, obtained using a computer-assisted personal interviewing technique once a year during notified weekdays, which takes ~1 h to complete. The individuals in the sample are interviewed regarding individual healthcare utilisation, health expenditure, sociodemographic characteristics, lifestyle, and health-related factors. Although the KHP survey started in 2008, in this study, data from 2014 to 2018 are utilised because of changes introduced in the dataset on information concerning chronic diseases from 2014.

From a total of 72,867 observations of individuals aged 19 years and above, this study excluded cases involving non-reporting of information regarding unmet healthcare needs and explanatory variables. Therefore, the final study sample consisted of an unbalanced panel sample comprising 68,930 observations (31,838 from men and 37,092 from women; 94.6%) of 16,535 individuals (7,864 men and 8,671 women), with an average of 4.17 observations per individual (standard deviation = 1.40, range = 1–5).

### Measurements

#### Outcome Variable

An individual's experience of having a non-financial or financial barrier to healthcare was determined by their answers to two questions from the KHP survey: “Was there at least one type of medical care or examination (other than dental care and examination) during the last year (12 months) that you did not receive although you needed it?” and, for the individuals who answered “yes”, the accompanying question was: “Among the following, what was the major reason for not receiving the needed medical care or examination?” The reasons were listed as follows: (1) Financial reasons (burden of medical expenditure); (2) Health facilities were too far away; (3) It was difficult to visit a healthcare facility due to either functional limitation or poor health; (4) I had no one to take care of the children; (5) I felt my symptom was not severe; (6) I had no information on where to go; (7) I had no time to visit a healthcare facility; (8) I could not make a reservation at a proper time; (9) I had no regular doctor; (10) Other reasons.

Individuals who answered “yes” to the first question indicated that they had failed in meeting their healthcare needs due to a barrier to healthcare. This study categorised all respondents into three groups, namely, no barrier, financial barrier, and non-financial barrier groups. The individuals who answered “no” to the first question were categorised into the no barrier group; those individuals who answered “yes” to the first question and who chose the financial reasons option in the second question were categorised into the financial barrier group and those who answered “yes” to the first question and who chose any one of the listed reasons apart from financial reasons for the second question were categorised into the non-financial barrier group.

#### Explanatory Variables

The explanatory variables consisted of sociodemographic characteristics as well as physical and health conditions. The sociodemographic characteristics were as follows: gender (men and women); age; marital status (married and non-married, where non-married included never married, separated, widowed, or divorced); residential area (Seoul metropolitan area, including Seoul, Incheon, and Gyeonggi province; and areas outside of the Seoul metropolitan area); the highest level of formal education completed (lower than college, and college, or higher); occupation (no job, blue-collar job, and white-collar job, where no job included the unemployed and individuals outside of economic activity, such as house-keepers, students, and retired individuals); household income (lowest, medium, and highest quintile, where for each wave, household income was adjusted for household size using the square root's equivalence scale, and the medium included the three middle quintiles) (16); status of public financially secured protection programmes (NHI and MCA programmes); status of private health insurance (“yes” or “no”, indicating whether an individual is a beneficiary of at least one private health insurance plan).

Physical and health conditions were as follows: functional limitation (“yes” or “no”); current smoker (“yes” or “no”); alcohol consumer (“yes” or “no”); active routine of physical exercise activity (“yes” or “no”); obese (“yes” or “no”); poor self-assessed health (“yes” or “no”); number of chronic diseases (none, one to three, or four or more); hypertension (“yes” or “no”); diabetes mellitus (“yes” or “no”); and dyslipidaemia (“yes” or “no”). Functional limitation was based on an individual's answer to the question: “Is your routine of daily living (conducting work, housekeeping, study, and social, leisure, or familiar activities) limited due to a disease or an injury?” An active routine of physical exercise activity was defined based on an individual's answers when assessing their engagement in any three kinds of physical exercise (walking, medium-level, or high-level exercise) for 30 min or longer at least thrice a week. Based on an individual's answer to questions on height and weight, obesity was defined in terms of an individual's body mass index being at least 25 kg/m^2^, which is in line with the recommendation in the Asia-Pacific criteria concerning obesity status provided by the World Health Organisation Western Pacific Region (17). Poor self-assessed health involved an individual's self-rating of their general health as “poor” or “very poor” among the options of “excellent, very good, fair, poor, or very poor”. Chronic disease was determined based on self-reported answers on whether an individual was suffering from any chronic disease diagnosed by a physician at the time of the survey.

### Statistical Analyses

In conducting this study, it was considered that comparing the percentage of people experiencing non-financial or financial barriers to healthcare across European countries with those in Korea would further advance the understanding on this matter. To undertake the comparison, in this study, data derived from the European Union Statistics on Income and Living Conditions (EU-SILC) provided by the statistical office of the European Union (EUROSTAT) (9) for 37 European countries and the analysed data collected from the KHP survey were used. The EU-SILC contains data on individuals' experiences of having unmet medical care needs in the 12 months prior to providing such information and their major reason for their unmet healthcare needs each year since 2008. The data obtained concerned individuals aged 16 years and above in European Union member states, European Economic Area countries, and Switzerland. As the COVID-19 pandemic began in late 2019 and is very likely to have influenced health service utilisation worldwide, this study selected 2018 as the reference year.

When exploring the characteristics associated with non-financial or financial barriers to healthcare in Korea, three types of barriers to healthcare (no barrier, financial barrier, and non-financial barrier) were observed. A mixed multinomial logit model was utilised to analyse the panel data. This model is known to relax the assumption of the independence of irrelevant alternative property of conventional logit and probit models for polychotomous choice situations (18–20).

First, the distributions of the three types of barriers to healthcare each year were examined and, on determining that the distributions differed between genders for each year (*p* < 0.05), all the analyses were stratified by gender. Second, for multivariable analysis, each of the explanatory variables was continually re-categorised and their reference categories were also redefined, with the age variable centred around the median (53). Consequently, the model did not exhibit any considerable multicollinearity, with the value of the variance inflation factor being <3.32 for each gender.

Third, it was deemed to be difficult to understand how an individual's probability of experiencing each type of barrier to healthcare varies across individual characteristics based only on the results obtained from the mixed multinomial logit model. Therefore, this study employed the average marginal effects method (18) and computed the average adjusted probability (AAP) that an individual with a particular characteristic would experience each of the three types of barriers to healthcare, with all other characteristics of the individual being the same, and estimated the 95% confidence intervals of those AAPs. In addition, for ease of understanding, this study depicted the changes in the AAPs across different age groups by gender with the help of visual curves. All characteristics were considered time varying (i.e., could change with time). Statistical analyses were performed using SAS 9.4 software (SAS Institute, Cary, NC, USA) and STATA 17 software (StataCorp, College Station, TX, USA).

## Results

### Percentage of People Experiencing Each Type of Barrier to Healthcare Across Selected Countries

**Figure 1:**
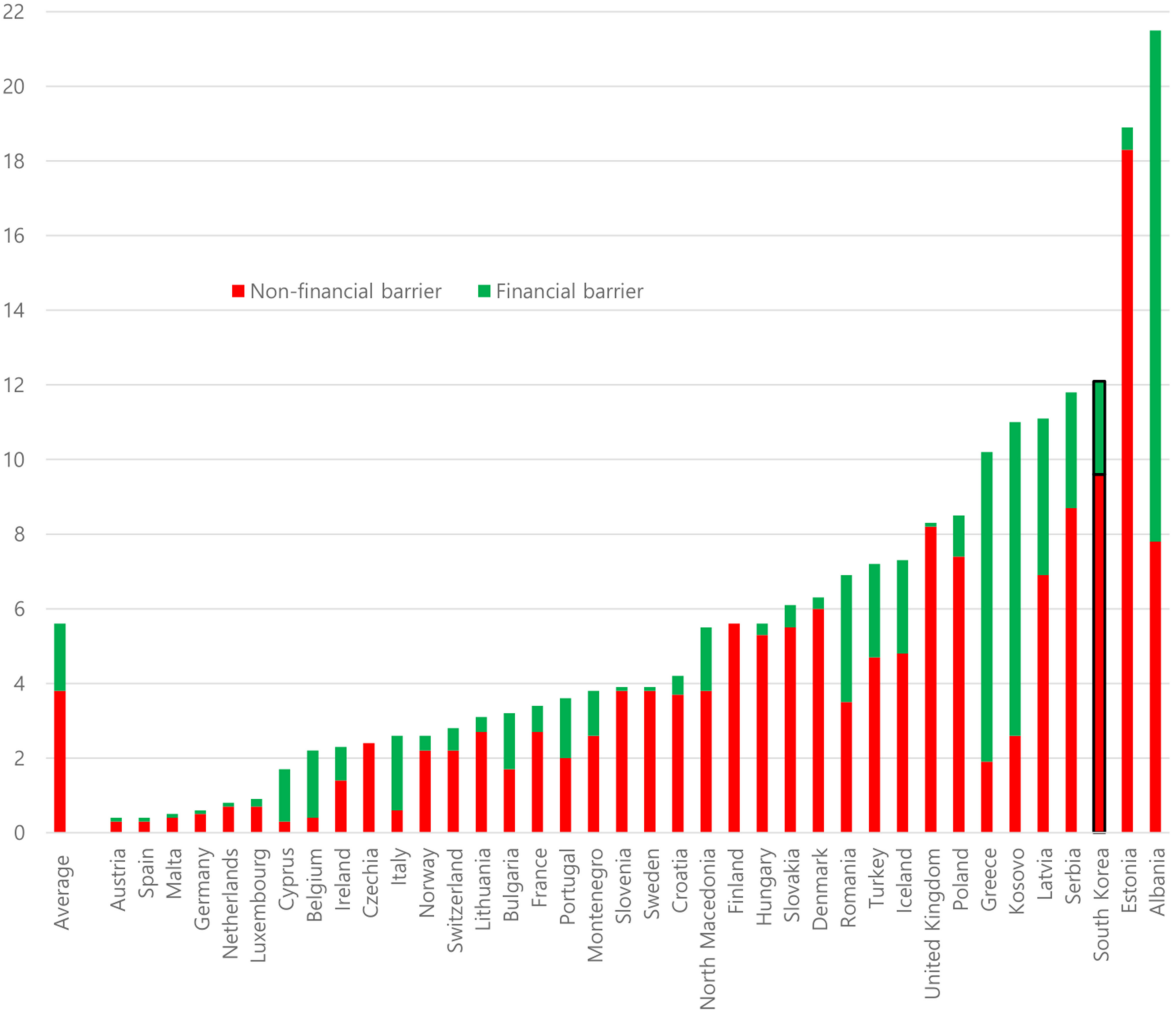
Percentage of people experiencing either non-financial or financial barriers in 2018 across selected countries. Unmet needs for medical examination or treatment for multiple reasons in the previous 12 months. Individuals included those aged 16 years and above (37 European countries) and individuals aged 19 years and above (South Korea). Sources: EUROSTAT Database (EU-SILC, 2018) and the Korea Health Panel survey data (2018).

In 2018, the percentage of people experiencing a barrier to healthcare leading to unmet healthcare needs in the past 12 months was, on average, 5.6% for the 38 countries considered in this study including Korea (Figure 1).

This total percentage ranged from 0.4% in Austria and Spain to 21.5% in Albania. The percentage of people experiencing a non-financial barrier was found to be much higher than that of people experiencing a financial barrier. The percentage of people experiencing a non-financial barrier was, on average, 3.8%, ranging from 0.3% in Austria, Cyprus, and Spain to 18.3% in Estonia. By contrast, the percentage of people experiencing a financial barrier was, on average, 1.8%, ranging from 0.1% or lower in Austria, Czechia (the Czech Republic), Finland, Germany, Malta, the Netherlands, Slovenia, Spain, Sweden, and the UK to 13.7% in Albania. In the case of Korea, the percentage of people experiencing a barrier generally was 12.1%, of which 9.6% involved a non-financial barrier and 2.5% involved a financial barrier. Notably, each of the three percentages varied markedly across the 38 countries and, on average, non-financial barriers appeared to lead to unmet healthcare needs among individuals more often than financial barriers.

### Percentage of People Experiencing Each Type of Barrier to Healthcare by Gender in Korea

In 2018, 12.1% of the individuals in the sample (11.0% in men; 13.0% in women) reported that they had experienced a barrier leading to unmet healthcare needs in the last 12 months. The percentage of people experiencing a non-financial barrier was much higher than that of those experiencing a financial barrier (9.6 vs. 2.5% in total; 9.0 vs. 2.0% in men; 10.2 vs. 2.8% in women). The percentage of people experiencing a barrier leading to unmet healthcare needs between 2014 and 2018 was 14.1%, of which 10.1% involved a non-financial barrier and 4.0% involved a financial barrier. Clear gender differences were revealed in terms of experiencing a barrier, a non-financial barrier, or a financial barrier (12.5, 9.3, and 3.2%, respectively, among men vs. 15.4, 10.8, and 4.6%, respectively, among women). The sample characteristics and descriptive statistics for each year between 2014 and 2018 are displayed in Table 1 for men and Table 2 for women.

**Table 1:** Sample characteristics and their descriptive statistics for each year among men.

**Characteristics**	**2014 (*****n*** **=** **6,842)**		**2015 (*****n*** **=** **6,494)**		**2016 (*****n*** **=** **6,196)**		**2017 (*****n*** **=** **6,164)**		**2018 (*****n*** **=** **6,142)**	
	**Mean**	**SD**	**Mean**	**SD**	**Mean**	**SD**	**Mean**	**SD**	**Mean**	**SD**
Barrier to healthcare	0.112	(0.004)	0.123	(0.004)	0.097	(0.004)	0.096	(0.004)	0.110	(0.004)
Non-financial barrier	0.084	(0.003)	0.091	(0.004)	0.073	(0.003)	0.076	(0.003)	0.090	(0.004)
Financial barrier	0.028	(0.002)	0.032	(0.002)	0.024	(0.002)	0.020	(0.002)	0.020	(0.002)
**Sociodemographics**										
Age (years)	50.781	(0.204)	51.407	(0.212)	52.094	(0.219)	52.332	(0.223)	52.564	(0.227)
Non-married	0.266	(0.005)	0.271	(0.006)	0.274	(0.006)	0.284	(0.006)	0.293	(0.006)
Seoul metropolitan area	0.381	(0.006)	0.379	(0.006)	0.376	(0.006)	0.379	(0.006)	0.380	(0.006)
College or higher	0.355	(0.006)	0.361	(0.006)	0.368	(0.006)	0.377	(0.006)	0.385	(0.006)
**Occupation**										
No job	0.272	(0.005)	0.294	(0.006)	0.283	(0.006)	0.281	(0.006)	0.277	(0.006)
Blue-collar job	0.510	(0.006)	0.494	(0.006)	0.502	(0.006)	0.501	(0.006)	0.502	(0.006)
White-collar job	0.218	(0.005)	0.213	(0.005)	0.215	(0.005)	0.218	(0.005)	0.221	(0.005)
**Household income**										
Lowest quintile	0.172	(0.005)	0.175	(0.005)	0.169	(0.005)	0.166	(0.005)	0.164	(0.005)
Medium	0.615	(0.006)	0.613	(0.006)	0.619	(0.006)	0.621	(0.006)	0.618	(0.006)
Highest quintile	0.213	(0.005)	0.212	(0.005)	0.213	(0.005)	0.213	(0.005)	0.218	(0.005)
Medical care aid	0.025	(0.002)	0.027	(0.002)	0.030	(0.002)	0.029	(0.002)	0.029	(0.002)
Private health insurance	0.669	(0.006)	0.697	(0.006)	0.711	(0.006)	0.728	(0.006)	0.740	(0.006)
**Physical and health conditions**										
Functional limitation	0.052	(0.003)	0.063	(0.003)	0.057	(0.003)	0.050	(0.003)	0.053	(0.003)
Current smoker	0.414	(0.006)	0.367	(0.006)	0.367	(0.006)	0.354	(0.006)	0.345	(0.006)
Alcohol consumer	0.784	(0.005)	0.780	(0.005)	0.776	(0.005)	0.781	(0.005)	0.782	(0.005)
Active routine of physical exercise activity	0.409	(0.006)	0.419	(0.006)	0.430	(0.006)	0.413	(0.006)	0.403	(0.006)
Obese	0.281	(0.005)	0.286	(0.006)	0.294	(0.006)	0.321	(0.006)	0.323	(0.006)
Poor self-assessed health	0.128	(0.004)	0.105	(0.004)	0.112	(0.004)	0.109	(0.004)	0.114	(0.004)
**Number of chronic diseases**										
None	0.420	(0.006)	0.418	(0.006)	0.405	(0.006)	0.420	(0.006)	0.418	(0.006)
One to three	0.434	(0.006)	0.429	(0.006)	0.433	(0.006)	0.436	(0.006)	0.420	(0.006)
Four or more	0.146	(0.004)	0.153	(0.004)	0.163	(0.005)	0.145	(0.004)	0.163	(0.005)
Hypertension	0.230	(0.005)	0.239	(0.005)	0.246	(0.005)	0.239	(0.005)	0.243	(0.005)
Diabetes mellitus	0.094	(0.004)	0.100	(0.004)	0.108	(0.004)	0.105	(0.004)	0.111	(0.004)
Dyslipidemia	0.086	(0.003)	0.103	(0.004)	0.119	(0.004)	0.121	(0.004)	0.136	(0.004)

*SD denotes standard deviation. Source: The Korea Health Panel survey data (2014–2018)*.

**Table 2:** Sample characteristics and their descriptive statistics for each year among women.

**Characteristics**	**2014 (*****n*** **=** **7,849)**		**2015 (*****n*** **=** **7,507)**		**2016 (*****n*** **=** **7,275)**		**2017 (*****n*** **=** **7,244)**		**2018 (*****n*** **=** **7,217)**	
	**Mean**	**SD**	**Mean**	**SD**	**Mean**	**SD**	**Mean**	**SD**	**Mean**	**SD**
Barrier to healthcare	0.141	(0.004)	0.150	(0.004)	0.123	(0.004)	0.115	(0.004)	0.130	(0.004)
Non-financial barrier	0.103	(0.003)	0.106	(0.004)	0.086	(0.003)	0.084	(0.003)	0.102	(0.004)
Financial barrier	0.038	(0.002)	0.044	(0.002)	0.037	(0.002)	0.031	(0.002)	0.028	(0.002)
**Sociodemographics**										
Age (years)	52.343	(0.198)	53.024	(0.205)	53.718	(0.210)	54.217	(0.213)	54.643	(0.216)
Non-married	0.347	(0.005)	0.355	(0.006)	0.366	(0.006)	0.373	(0.006)	0.383	(0.006)
Seoul metropolitan area	0.377	(0.005)	0.373	(0.006)	0.372	(0.006)	0.373	(0.006)	0.372	(0.006)
College or higher	0.263	(0.005)	0.264	(0.005)	0.267	(0.005)	0.276	(0.005)	0.285	(0.005)
**Occupation**										
No job	0.505	(0.006)	0.525	(0.006)	0.510	(0.006)	0.503	(0.006)	0.487	(0.006)
Blue-collar job	0.330	(0.005)	0.305	(0.005)	0.316	(0.005)	0.321	(0.005)	0.332	(0.006)
White-collar job	0.165	(0.004)	0.170	(0.004)	0.174	(0.004)	0.176	(0.004)	0.180	(0.005)
**Household income**										
Lowest quintile	0.226	(0.005)	0.222	(0.005)	0.227	(0.005)	0.229	(0.005)	0.231	(0.005)
Medium	0.586	(0.006)	0.589	(0.006)	0.584	(0.006)	0.588	(0.006)	0.586	(0.006)
Highest quintile	0.189	(0.004)	0.189	(0.005)	0.189	(0.005)	0.183	(0.005)	0.183	(0.005)
Medical care aid	0.037	(0.002)	0.038	(0.002)	0.040	(0.002)	0.037	(0.002)	0.036	(0.002)
Private health insurance	0.683	(0.005)	0.712	(0.005)	0.725	(0.005)	0.737	(0.005)	0.748	(0.005)
**Physical and health conditions**										
Functional limitation	0.065	(0.003)	0.092	(0.003)	0.079	(0.003)	0.069	(0.003)	0.073	(0.003)
Current smoker	0.027	(0.002)	0.021	(0.002)	0.022	(0.002)	0.023	(0.002)	0.021	(0.002)
Alcohol consumer	0.523	(0.006)	0.548	(0.006)	0.545	(0.006)	0.543	(0.006)	0.548	(0.006)
Active routine of physical exercise activity	0.360	(0.005)	0.372	(0.006)	0.360	(0.006)	0.343	(0.006)	0.310	(0.005)
Obese	0.214	(0.005)	0.217	(0.005)	0.219	(0.005)	0.230	(0.005)	0.228	(0.005)
Poor self-assessed health	0.187	(0.004)	0.173	(0.004)	0.181	(0.005)	0.186	(0.005)	0.185	(0.005)
**Number of chronic diseases**										
None	0.334	(0.005)	0.326	(0.005)	0.321	(0.005)	0.341	(0.006)	0.330	(0.006)
One to three	0.416	(0.006)	0.419	(0.006)	0.410	(0.006)	0.408	(0.006)	0.400	(0.006)
Four or more	0.250	(0.005)	0.254	(0.005)	0.269	(0.005)	0.250	(0.005)	0.270	(0.005)
Hypertension	0.251	(0.005)	0.260	(0.005)	0.267	(0.005)	0.264	(0.005)	0.268	(0.005)
Diabetes mellitus	0.092	(0.003)	0.096	(0.003)	0.101	(0.004)	0.099	(0.004)	0.103	(0.004)
Dyslipidemia	0.124	(0.004)	0.149	(0.004)	0.170	(0.004)	0.172	(0.004)	0.191	(0.005)

*SD denotes standard deviation. Source: The Korea Health Panel survey data (2014–2018)*.

### Average Adjusted Probability of Experiencing Each Type of Barrier Across Characteristics by Gender in Korea

**Figure 2:**
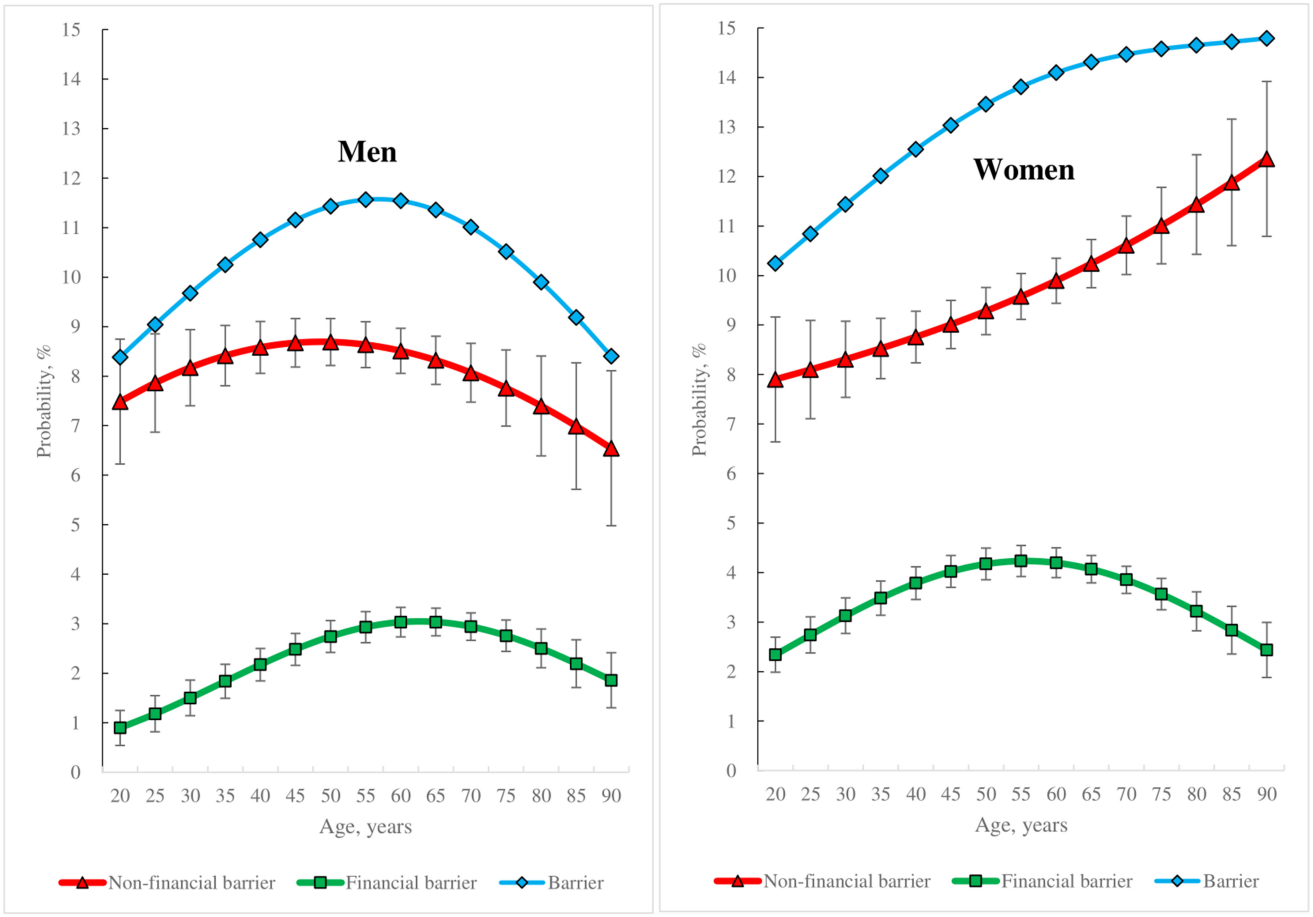
Gender-specific differences in the average adjusted probabilities of experiencing non-financial or financial barriers that led to unmet healthcare needs across different age groups and the 95% confidence intervals.

Except for obesity, all characteristics under investigation were associated with the AAP of experiencing non-financial or financial barriers at a significance level of 0.1 or less (Table 3; Figure 2).

**Table 3:** The average adjusted probabilities of experiencing non-financial or financial barriers that led to unmet healthcare needs.

**Characteristics**	**Men (*****n*** **=** **31,838)**			**Women (*****n*** **=** **37,092)**		
	**Non-financial barrier (*N*)**	**Financial barrier (*F*)**	**Barrier (*N* + *F*)**	**Non-financial barrier (*N*)**	**Financial barrier (*F*)**	**Barrier (*N* + *F*)**
All	8.3^***^	2.5^***^	10.8	9.6^***^	3.6^***^	13.2
Married (*R*)	8.6	2.3	10.9	9.3	3.0	12.3
Non-married	7.5^**^	3.1^***^	10.6	10.1^**^	4.4^***^	14.6
The other areas (*R*)	8.0	2.1	10.1	9.4	3.1	12.5
Seoul metropolitan area	8.7^**^	3.4^***^	12.1	9.9	4.7^***^	14.6
Lower than college (*R*)	8.3	2.6	11.0	9.3	3.7	13.0
College or higher	8.3	2.0^***^	10.2	10.6^***^	2.7^***^	13.3
No job (*R*)	5.9	2.7	8.6	7.8	3.7	11.5
Blue-collar job	9.6^***^	2.4	12.0	11.6^***^	3.6	15.2
White-collar job	8.6^***^	1.9^**^	10.5	11.7^***^	2.9^*^	14.7
Household income, lowest quintile (*R*)	8.7	5.0	13.7	9.4	6.3	15.7
Household income, medium	8.4	1.8^***^	10.2	9.9	2.5^***^	12.5
Household income, highest quintile	7.9	0.6^***^	8.5	9.4	0.7^***^	10.1
National health insurance (*R*)	8.2	2.5	10.7	9.5	3.5	13.1
Medical care aid	11.0^**^	2.8	13.8	11.8^**^	4.2^**^	16.0
Private health insurance, no (*R*)	8.0	2.7	10.7	9.4	3.9	13.3
Private health insurance, yes	8.4	2.3^**^	10.7	9.7	3.4^**^	13.1
Functional limitation, no (*R*)	7.8	2.3	10.1	9.0	3.3	12.3
Functional limitation, yes	17.8^***^	3.7^***^	21.5	17.4^***^	4.9^***^	22.3
Current smoker, no (*R*)	7.5	2.3	9.8	9.5	3.5	13.1
Current smoker, yes	9.6^***^	2.9^***^	12.4	12.5^***^	5.4^***^	17.8
Alcohol consumer, no (*R*)	7.8	2.5	10.3	8.8	3.6	12.4
Alcohol consumer, yes	8.4	2.5	10.9	10.4^***^	3.6	14.0
Active routine of physical exercise activity, no (*R*)	8.6	2.5	11.0	9.9	3.6	13.5
Active routine of physical exercise activity, yes	7.9^**^	2.6	10.5	9.0^***^	3.6	12.6
Obese, no (*R*)	8.3	2.5	10.8	9.5	3.5	13.1
Obese, yes	8.4	2.4	10.7	9.8	3.8	13.6
Poor self-assessed health, no (*R*)	7.7	2.0	9.6	8.6	2.6	11.2
Bad self-assessed health, yes	13.5^***^	4.5^***^	17.9	14.6^***^	5.8^***^	20.4
Number of chronic diseases, none (*R*)	7.8	1.7	9.5	9.7	2.0	11.7
One to three chronic diseases	9.1^***^	2.7^***^	11.8	10.2	3.5^***^	13.7
Four or more chronic diseases	7.4	2.8^***^	10.2	8.9	4.3^***^	13.2
Hypertension, no (*R*)	8.4	2.7	11.1	9.9	3.8	13.7
Hypertension, yes	7.9	2.2^***^	10.1	8.8^**^	3.4^**^	12.2
Diabetes mellitus, no (*R*)	8.3	2.5	10.8	9.7	3.7	13.4
Diabetes mellitus, yes	8.0	2.4	10.4	9.0	3.3^*^	12.2
Dyslipidemia, no (*R*)	8.4	2.5	10.9	9.7	3.6	13.4
Dyslipidemia, yes	7.3^***^	2.4	9.6	9.1	3.5	12.6

*Results of an average marginal effects analysis. R denotes reference category*.

*** *p < 0.01*.

** *p < 0.05*.

* *p < 0.1*.

*Source: The Korea Health Panel survey data (2014–2018)*.

The AAP of experiencing a non-financial barrier was higher than that of experiencing a financial barrier in both men (8.3 vs. 2.5%) and women (9.6 vs. 3.6%). However, both AAPs were higher in women than in men (Table 3). Both AAPs varied across age groups and between genders (Figure 1). The AAP of experiencing a financial barrier increased and then decreased with age in both men and women. However, the AAP of experiencing a non-financial barrier increased and then decreased with age in men, whereas the AAP continued to increase with age in women.

Men living in the Seoul metropolitan area showed higher values in the AAPs of experiencing both non-financial and financial barriers than those living outside (8.7 vs. 8.0%; 3.4 vs. 2.1%) (Table 3). In contrast, women living in the Seoul metropolitan area showed higher values in the AAPs of experiencing a financial barrier than those living outside (4.7 vs. 3.1%). College graduates had a lower value in the AAP of experiencing a financial barrier than individuals with a lower educational level in both men (2.0 vs. 2.6%) and women (2.7 vs. 3.7%). However, regarding the AAP of experiencing a non-financial barrier, women college graduates showed a higher value than women with a lower educational level (10.6 vs. 9.3%).

Job-holders had a higher value in the AAP of experiencing a non-financial barrier in both men and women compared to the jobless category (in men, 9.6% having a blue-collar job, 8.6% having a white-collar job, and 5.9% in the jobless category; in women, 11.7% having a white-collar job, 11.6% having a blue-collar job, and 7.8% in the jobless category). The AAP of experiencing a financial barrier in individuals having a white-collar job exhibited a lower value than the jobless category in both men (1.9 vs. 2.7%) and women (2.9 vs. 3.7%). When household income was considered, the AAP of experiencing a financial barrier decreased with increasing household income in both men and women (the AAPs for individuals belonging to the lowest quintile, the medium category, and the highest quintile were 5.0, 1.8, and 0.6% in men, respectively, and 6.3, 2.5, and 0.7% in women, respectively). Compared to individuals covered by the NHI programme, those covered by the MCA programme showed higher values in the AAP of experiencing a non-financial barrier in men (8.2 vs. 11.0%) and in the AAPs of experiencing both a non-financial barrier (9.5 vs. 11.8%) and a financial barrier (3.5 vs. 4.2%) in women.

Individuals having a functional limitation, relative to individuals with no functional limitation, exhibited an exceedingly higher value in the AAP of experiencing a non-financial barrier in both men (17.8 vs. 7.8%) and women (17.4 vs. 9.0%) as well as a higher value in the AAP of experiencing a financial barrier in both men (3.7 vs. 2.3%) and women (4.9 vs. 3.3%). The AAPs of experiencing both non-financial and financial barriers were higher in current smokers than in current non-smokers in both men and women (for experiencing a non-financial barrier, 9.6 vs. 7.5% in men and 12.5 vs. 9.5% in women; and for experiencing a financial barrier, 2.9 vs. 2.3% in men and 5.4 vs. 3.5% in women).

Individuals who reported that their health was poor exhibited higher values in the AAPs of experiencing both non-financial and financial barriers compared to individuals reporting that their health was not poor in both men and women (for a non-financial barrier, 13.5 vs. 7.7% in men and 14.6 vs. 8.6% in women; for a financial barrier, 4.5 vs. 2.0% in men and 5.8 vs. 2.6% in women).

Individuals having one to three chronic diseases had a higher value in the AAP of experiencing a non-financial barrier than individuals having no chronic disease only in men (7.8 vs. 9.1%). However, the AAP of experiencing a financial barrier seemed to increase with the number of chronic diseases in both men and women (for no chronic disease, one to three chronic diseases and four and more chronic diseases, 1.7, 2.7, and 2.8% in men, respectively, and 2.0, 3.5, and 4.3% in women, respectively).

## Discussion

### Differences in the Percentage of People Experiencing Each Type of Barrier to Healthcare Between European Countries and Korea

Based on the results of the present study concerning 37 European countries and Korea, an average of 5.6% of individuals reported not receiving a medical examination or treatment that they needed in the past 12 months due to either a non-financial barrier (3.8%) or a financial barrier (1.8%). The findings indicate that the percentage of people experiencing a non-financial barrier comprised ~70% of the percentage of people experiencing a barrier that led to unmet healthcare needs and was more than twice as high as that of people experiencing a financial barrier. Individuals seemed not to be able to obtain timely and adequate healthcare when they needed it more often due to a non-financial barrier than a financial barrier in Korea and in most European countries, except for Albania, Belgium, Cyprus, Greece, Italy, and Kosovo.

Compared to individuals in the European countries, individuals' access to necessary healthcare in Korea seemed to be more severely hampered by a high prevalence of non-financial barriers. Based on the study results, the percentage of people experiencing a non-financial barrier (9.6%) was ~4 times higher than that of experiencing a financial barrier (2.5%). Therefore, it is necessary for policy-makers in Korea to reform the nation's healthcare system to address these non-financial barriers along with concurrent efforts to reduce financial barriers.

To examine the specific barriers to healthcare that led to unmet healthcare needs in Korea, the present study conducted a further estimation of the prevalence rates of each specific barrier using domain analysis with survey weights. The results showed that in 2018, for adults aged 19 years and above, the percentage of individuals who experienced a barrier leading to unmet healthcare needs was 11.7% [standard error (SE) = 0.3], the percentage of individuals who experienced a non-financial barrier was 9.5% (SE = 0.3), and the percentage of individuals who experienced a financial barrier was 2.1% (SE = 0.1). Concerning a non-financial barrier, the highest rate of prevalence was shown in terms of: “7) I had no time to visit a healthcare facility” (4.7%, SE = 0.2), followed by “5) I felt my symptom was not severe” (3.4%, SE = 0.2), and “1) Financial reasons (burden of medical expenditure)” (2.1%, SE = 0.1). These three barriers (10.2%) accounted for most (87.2%) of the barriers to healthcare (11.7%) in Korea (detailed results can be provided on request).

To compare the percentage of people experiencing each barrier to healthcare between the European countries and Korea, the present study further calculated the average value of each barrier in 37 European countries in 2018 using data from the EUROSTAT database. The results showed that the most severe barrier to healthcare for individuals in European countries was a financial barrier, as demonstrated through the following factors identified by respondents: “too expensive” (1.7%), followed by “waiting list” (1.3%), and “wanted to wait and see if the problem got better on its own” (0.8%). These three barriers (3.3%) accounted for most (71.7%) of the barriers to healthcare (5.3%) in the European countries.

Based on the above results, Korea's healthcare system would appear to require differing policies to be implemented compared with the European countries to provide its population with timely and adequate healthcare facilities. Reforms needed in Korea would include: (1) helping busy individuals receive healthcare when they need it, (2) assisting individuals to make a quick decision if their health problem is serious enough to require them to see a physician, and (3) aiding individuals who cannot afford to pay out-of-pocket to address their health issues.

### Differences Between the Characteristics Associated With Non-financial Barriers and Those Associated With Financial Barriers in Korea

Identifying the specific characteristics of individuals that are associated with their experience of non-financial and financial barriers is pivotal for healthcare policy-makers. Using this information, the Korean government could identify priority targets to reduce barriers to healthcare.

Previous studies have documented that women experience more unmet healthcare needs than men (21, 22). This study also considered the gender dimension and found that women tended to have higher risks of experiencing non-financial and financial barriers than men. On further analysis of these gender differences in relation to barriers to healthcare needs, this study conducted a calculation using the results displayed in Table 3 and Figure 2, in which the AAP in men was distinguished from the AAP in women and the excess values for the AAP of experiencing each barrier in women relative to men were obtained in terms of percentage points. Based on the results, for non-financial barriers, the largest excess values of the AAP were shown in individuals aged 90 years (5.8% points), followed by individuals aged 85 years (4.9% points), and individuals aged 80 years (4.0% points). For financial barriers, the largest excess values of the AAP were exhibited in current smokers (2.5% points), followed by individuals aged 25, 30, 35, and 40 years (1.6% points for all four age groups, respectively). This finding suggests that age is an important characteristic in explaining gender differences in the risk of experiencing each barrier after adjusting the other characteristics. Specifically, to reduce the risk of experiencing each barrier in women relative to men, it is recommended that the Korean government prioritise the healthcare needs of women aged 80 years and above to help address their non-financial barriers, and the needs of women aged 40 years and under to help address their financial barriers.

Previous studies have shown a decrease in unmet healthcare needs with increasing age (23), mainly because older individuals care more about their health conditions and visit a physician more often (24). Several studies on barriers to healthcare have shown that system barriers, including financial barriers, increase with age (25), whereas personal factors, indicated through comments such as: “I had no time to visit a healthcare facility”, decrease with age (25–28). However, based on the present study's findings using panel data and stratifying the analysis by gender, the association between age and the risk of experiencing a barrier leading to unmet healthcare needs differed between genders in Korea, as shown in Figure 2. In men, the risk of experiencing a barrier changed with age, shown by the bell curve, with the risk of experiencing a non-financial and a financial barrier also exhibiting this pattern; but in women, while the risk of experiencing a barrier increased at a diminishing rate with age, the risk of experiencing a financial barrier changed in a bell curve shape with age, and the risk of experiencing a non-financial barrier rose at an increasing rate with age. In addition, the present study found that individuals of a particular age group, that is, men ~60 years old and women ~55 years old, had the highest risk of experiencing a financial barrier among all the age groups. The age groups with the highest risk of experiencing a non-financial barrier comprised men aged between 45 and 50 years and women aged 90 years and above. These findings suggest that healthcare policies to protect individuals from both non-financial and financial barriers to healthcare should be customised across age groups and between genders in Korea.

While the influence of residential area on access to healthcare depends on how a rural area is defined (29), previous studies have reported that individuals in rural areas generally tend to have a higher rate of unmet healthcare needs due to poor roads leading to healthcare facilities and sparsely located facilities (27, 30). However, the current study shows that both men and women living in the Seoul metropolitan area were exposed to a greater risk of a financial barrier after adjusting for other characteristics than those living outside this area. This finding may be related to the fact that healthcare providers are highly concentrated in the Seoul metropolitan area, which may intensify competition-driven physician-induced demand (31, 32). It may be postulated that that these healthcare providers offer high-quality and high-price healthcare services not covered by the NHI programme or that they are likely to advise individuals to visit them more often, which many individuals in the Seoul metropolitan area cannot afford to pay for. Future research needs to explore whether an increased risk of experiencing a financial barrier to healthcare in the Seoul metropolitan area arises from a higher burden of co-payments for healthcare services that are not covered by the NHI programme or from more frequent visits to healthcare facilities that are covered by the NHI programme. Regarding non-financial barriers, the study found that the risk of experiencing a non-financial barrier was higher in women living in the Seoul metropolitan area than in those living outside. It might be the case that compared to women living outside, women living in the Seoul metropolitan area are more likely to face severe time constraints as they often tend to be engaged in jobs in the labour market in addition to traditional duties, such as housekeeping, child-rearing, and caring for parents (33–35).

It has been generally acknowledged that individuals within a lower socioeconomic category face more barriers to healthcare. However, in terms of the relation between education and unmet healthcare needs, past findings are mixed, showing a negative relation in some studies (36) and a positive relation in others (37). The results of the current study indicate that tertiary education negatively correlates with experiencing a financial barrier in men and women. In contrast, tertiary education has a positive relation with experiencing a non-financial barrier only in women. This finding suggests that highly educated women may postpone medical visits or treatments in Korea, possibly due to time constraints, as described previously. Hence, it is proposed that policies to reduce each type of barrier leading to unmet healthcare needs need to be designed differently based on education level and gender.

Household income has long been considered a major risk factor for unmet healthcare needs, and an increasing number of studies have recommended policy action for minimising financial barriers to improve access to healthcare (27, 36, 38). However, the results of this study show that a higher level of household income reduces the risk of experiencing a financial barrier in both men and women but fails to reduce the risk of experiencing a non-financial barrier in both. This finding suggests that although financial barriers may be minimised through various policies, a considerable number of unmet healthcare needs are very likely to persist due to non-financial barriers, as shown in Figure 2. When job status is considered, this study found that jobless individuals, such as housekeepers, students, the unemployed, and the retired are exposed to a higher risk of facing a financial barrier but a lower risk of a non-financial barrier, when compared with job holders in the labour market. Based on this finding, a pertinent suggestion would be that policies for maximising access to necessary healthcare should be customised to distinguish between the jobless category and job holders. Recent studies have found that jobless women are exposed to an increased risk of mental health issues, and recommend well-designed policy options to cater to their needs (39, 40).

Various studies have shown that disability worsens the ability to access healthcare services (41–43). Individuals with disabilities often report that their healthcare needs are not attended to and that they feel abandoned (44). Women with disabilities experience additional barriers in accessing healthcare services more often than their male counterparts (22, 45). In one study, disabled individuals in the UK reported poorer access to healthcare, with the main barriers being difficulties in transportation, high cost, and long waiting lists (22). The present study found that individuals with a functional limitation exhibited a higher risk of experiencing a financial barrier and a much higher risk of experiencing a non-financial barrier in both men and women, with women being more vulnerable than men in both risk categories, compared to those with no functional limitation. These findings are of concern, given rapid population ageing in Korea and the increase in the number of individuals with a functional limitation (15). As such, an aged population is more likely to experience increased barriers in accessing necessary healthcare. A recent study involving 31 European countries found a considerable variation in the physical accessibility of primary healthcare across these countries, concluding that national healthcare policies should increase the physical accessibility of primary healthcare services to improve access to healthcare for individuals with a disability (46).

Many studies have revealed the positive effects of healthcare on health, which indicates that unmet healthcare needs are likely to be negatively related to self-assessed health (25, 47). Similar to these studies, the present study found that, relative to the individuals who reported that their health was not poor, those who reported that their health was poor had a higher risk of facing non-financial and financial barriers in both men and women. However, it is worth noting that this negative association between the risk of experiencing each type of barrier and self-assessed health may be due to reverse causation. For example, individuals who reported that their health was poor could be considered as being more likely to report that their healthcare needs were not met. As such, future research should investigate the cause-and-effect relationship between subjective unmet healthcare needs and subjective health outcomes.

### Identifying Target Groups by Gender to Minimise the Risk of Experiencing Each Type of Barrier in Korea

Identifying target groups by gender can help policy-makers focus effectively on reducing non-financial or financial barriers to healthcare or both. To aid this process and on the basis of the findings (Table 3; Figure 2), this study first compared the AAP of experiencing a particular barrier between individuals with all the characteristics and all individuals in each gender. Second, this study selected characteristics of individuals that were associated with a higher AAP of experiencing the particular barrier than the AAP for all individuals in the same gender. Third, the selected characteristics of these individuals were divided into three groups comprising characteristics of individuals who would benefit from policy interventions: (1) only reduce a non-financial barrier to healthcare; (2) only reduce a financial barrier to healthcare; and (3) reduce both non-financial and financial barriers to healthcare.

The characteristics of individuals (based on gender) who would benefit from policy interventions to only reduce a non-financial barrier to healthcare were, for men: being married; having a blue-collar job or a white-collar job; living in the Seoul metropolitan area; being in the medium group of household income; being the holder of a private health insurance plan; being an alcohol consumer; engaging in no active, routine physical exercise; being obese; and not having dyslipidaemia. For women, the characteristics were as follows: having a college education or higher; having a blue-collar job or a white-collar job; being in the medium group of household income; being a holder of a private health insurance plan; being an alcohol consumer; engaging in no active, routine physical exercise; having no chronic diseases; having one to three chronic diseases; and having no dyslipidaemia.

Next, the characteristics of individuals (based on gender) who would benefit from policy interventions to only reduce a financial barrier to healthcare, for men, were as follows: being non-married, not having a private health insurance plan; engaging in active, routine physical exercise; and having four or more chronic diseases. For women, the characteristics were as follows: having a lower education level than college; not having a job; being in the lowest quintile of household income; not having a private health insurance plan; and having four or more chronic diseases.

Third, the characteristics of individuals (based on gender) who would benefit from policy interventions to reduce both non-financial and financial barriers to healthcare, for men, were as follows: living in the Seoul metropolitan area; being in the lowest quintile of household income; being an MCA beneficiary; having a functional limitation; being a current smoker; having poor self-assessed health; having one to three chronic diseases; and having no hypertension. For women, the characteristics were as follows: being non-married; living in the Seoul metropolitan area; being an MCA beneficiary; having a functional limitation; being a current smoker; being obese; having poor self-assessed health; not having hypertension; and not having diabetes mellitus.

### More Policy Suggestions

Even if all the financial barriers to healthcare are eliminated, the unequal distribution of healthcare services in terms of non-financial barriers could potentially lead to unequal access to healthcare. Studies on the effect of implementing healthcare reforms in Massachusetts, USA, found that the effects of expanding insurance-based financial coverage on access to healthcare were indeterminate (48–50). Unlike primary care physicians in the USA, those in both Canada and the UK operating within a universal health insurance system have reported that their patients had fewer problems associated with financial barriers to healthcare but greater problems related to non-financial barriers (51).

Therefore, because individuals' access to necessary healthcare may be severely hampered by a high prevalence of non-financial barriers, policy-makers in Korea need to address issues arising in relation to non-financial barriers as well as financial barriers, both at central and regional levels of government. For example, at the central government level, it is recommended to allocate more financial resources towards healthcare and ensure that healthcare personnel are optimally educated and trained, with a special focus on advancing the quality of care, maintaining an appropriate level of cost of care, and taking accountability for providing ready access to healthcare for the whole population. In addition, the present study found that, irrespective of gender, the risk of experiencing a financial barrier to healthcare is the highest in individuals belonging to the lowest quintile of household income and the lowest in individuals belonging to the highest quintile of household income. As Korea's NHI programme is characterised by a high degree of income redistribution (52), the central government needs to more clearly recognise differences in the risk of experiencing a financial barrier to healthcare between wealthier and poorer sections of the population and to create robust policies to address the situation.

At the regional government level, it is recommended that regional administrations create, organise, and coordinate the provision of healthcare facilities in each region and provide necessary healthcare services to each resident in each region proportionately with the use of appropriate mechanisms for reducing the barriers to healthcare. Concerning non-financial barriers to healthcare, individuals with a functional limitation irrespective of gender were found to be at the highest risk, while jobless individuals had the lowest risk. Based on this finding, it is recommended that regional governments implement planning in regional healthcare systems that promotes the possibility of jobless individuals helping individuals with a functional limitation to meet their healthcare needs through cooperation with a healthcare provider (or a provider network).

At both central and regional government levels in Korea, policy-makers should prioritise efforts to address the most common type of non-financial barrier that leads to unmet healthcare needs, that is, the time barrier, demonstrated through comments such as: “I had no time to visit a healthcare facility”. This time constraint barrier needs to be reduced for the benefit of working populations with relatively inflexible schedules. One way this could be done would be to set up onsite workplace clinics for workers at medium-scale or large-scale workplaces (53, 54). For workers at small-scale workplaces, the provision of appropriate after-hours healthcare services (55–57), and telehealth services (58–60) could expand their opportunities to receive timely healthcare services.

The second most common type of non-financial barrier identified as leading to unmet healthcare needs in Korea is an information barrier, demonstrated through comments such as: “I felt my symptom was not so serious”. This issue could be addressed through policy implementations that increase the number of healthcare providers and that reorganise their roles to ensure the provision of pertinent health information to individuals. It needs to be noted that, although most advanced countries consider primary care as an essential element of universal health coverage (59, 61–65), Korea has long been inattentive to the importance of primary care, as shown through the substantial shortage of primary care physicians (15). Therefore, Korea needs to equip itself with an adequate number of primary care physicians and recognise their indispensable role in the nation's healthcare system through ensuring an appropriate level of training and of remuneration. At the same time, it is recommended that the scope of practise for nurse practitioners and physician assistants be expanded in clinical areas where physicians have long been in short supply (66–69) and that shared medical appointments be encouraged to allow more patients to receive routine primary care (70).

The benefits of regular access to a primary healthcare provider have been documented in numerous studies (71, 72). A recent study on primary healthcare reforms in Canada found that having regular access to a doctor reduced the risk of unmet healthcare needs (25). Encouraging individuals to have a regular primary healthcare provider seems a good way to not only reduce the most prevalent non-financial barriers to healthcare (time and information barriers) through fostering telehealth services or healthcare providers' or social service providers' visits to patients (73, 74), but also to lessen financial barriers through maintaining continuity, comprehensiveness, and coordination of care (75). If regular primary healthcare providers and social service providers combine to form a primary care provider network to cater to the healthcare needs of individuals in each area of a country, it would be possible for individuals to receive timely and adequate healthcare services when they need them. For example, through social services offered by the primary care provider network, individuals with functional limitations could visit a doctor and those socioeconomically disadvantaged would be more likely to be able to overcome financial barriers to healthcare (76–80).

Furthermore, it is recommended that periodic health surveys at both central and regional government levels be implemented to clarify the full range of healthcare challenges and the prevalence rates of all types of barriers to healthcare, with the relevant factors identified and addressed more effectively. It is strongly recommended that Korea, which has much to learn from the universal health insurance systems operating in European countries, collaborates with the European Union to minimise barriers to healthcare by conducting health surveys informed by approaches adopted in those countries and formulating effective healthcare policies thereafter.

### Strengths

To the best of the author's knowledge, this is the first study to identify gender-specific characteristics associated with non-financial and financial barriers to healthcare and quantify the effects of these characteristics, using a nationally representative longitudinal dataset, panel multinomial logit model analysis with time-varying covariates, and the average margins effects method. This study is also the first to compare non-financial and financial barriers to healthcare in Korea with those in many European countries using EUROSTAT statistics and a nationally representative dataset of Korea. This study highlights that, even though policy-makers in most countries have been striving to reduce financial barriers to healthcare and help individuals meet their healthcare needs, the often-ignored non-financial barriers to healthcare need to be considered to ensure effective healthcare. As for the generalisability of the research findings, the method used in the present study can be applied to other socio-cultural and national settings in which a universal health insurance system operates.

### Limitations

This study has some limitations. First, concerning the subjective unmet needs in the EU-SILC survey, the question only refers to unmet needs for a medical examination or a doctor consultation in Czechia, Slovenia, and Spain, and it refers to unmet needs for “severe” illnesses in Germany. This might have resulted in lower rates of unmet needs compared with other countries, most of which refer to both a medical examination and treatment (81).

Second, as the KHP survey was not based on clinical assessment, self-reported data, such as unmet needs and their reasons, might have involved recall bias, while self-reported height and weight data may have resulted in measurement errors. However, self-reported data have been recognised as very useful in analysis despite the lack of physicians' assessment data because individuals are often more aware of their healthcare needs even though they may not know the names of diseases and treatment methods as accurately as physicians; and because individuals can provide more precise responses as to why their healthcare needs were not met. Such self-reported data have been used in several studies on unmet healthcare needs in the USA (82, 83), Canada (23, 25), Europe (28, 84, 85), Asia (27, 86), and Africa (87).

Third, the KHP survey only asked respondents about one major reason for unmet healthcare needs in the last 12 months. Consequently, it was not possible to identify barriers that the respondents were able to overcome that allowed them to access healthcare. Future studies might seek to identify these types of barrier, which would be beneficial in research and for policy development by revealing how individuals succeed in overcoming barriers to healthcare. Moreover, because the survey sought information concerning one major reason for unmet healthcare needs, it was not possible to rank the relative importance of barriers that led to unmet healthcare needs. If data on the relative importance of barriers became available, researchers could conduct a more in-depth investigation including changes in the relative importance of barriers to healthcare over a lifetime.

Fourth, given the lack of related information, the present study could not consider other potential characteristics, such as social capital and social support (24, 88), and consumers' emotional satisfaction towards healthcare services (89). Future research needs to investigate whether these characteristics might be significant in determining the risk of barriers to healthcare in Korea.

Fifth, this study used a panel-data mixed multinomial logit model, which, irrespective of its various advantages, assumes that survey weights must be the same for all observations of a case. Therefore, this study could not apply survey weights for the main part of analysis because survey weights for this study's sample differ across individual people and across years. However, the results remain important in that this study did not intend to make statistics computed from the data more representative of the population but intended to reveal the importance of non-financial barriers to healthcare relative to financial barriers to healthcare.

Finally, although it was beyond the scope of this study, it would be of great interest to incorporate characteristics, such as ethnicity or immigrant status, into this type of analysis (26, 38). However, the KHP survey dataset does not include such information.

## Conclusions

Although financial barriers that lead to unmet healthcare needs have long been recognised as a pivotal risk factor in healthcare access, many individuals have experienced unmet healthcare needs due to a variety of non-financial barriers (5, 83, 90–93). The present study found notable differences across European countries and Korea in the percentage of people experiencing non-financial and financial barriers that led to unmet healthcare needs. Further, in most countries including Korea, compared to financial barriers, non-financial barriers more often prevent individuals from meeting their healthcare needs. Furthermore, the individual characteristics associated with experiencing non-financial barriers were found to differ from those associated with experiencing financial barriers. These findings indicate that, even after eliminating financial barriers, non-financial barriers could still generate a considerable degree of unmet healthcare needs and disparity among individuals.

Therefore, it is recommended that current universal health insurance systems apply targeted policy instruments to reduce the burden of non-financial barriers to healthcare to achieve effective universal health coverage. This recommendation would benefit countries such as Korea, where people experience unmet healthcare needs more often due to non-financial barriers than financial barriers, and which is facing the challenges of rapid population ageing and an increasingly higher prevalence of functional limitations.

## Data Availability Statement

Publicly available datasets were analysed in this study. This data can be found here: data are from the Korea Health Panel survey, which is available to the scientific community with a signed data access agreement from the Korea Institute for Health and Social Affairs and the National Health Insurance Service database (https://www.khp.re.kr:444/eng/main.do).

## Ethics Statement

This study used the secondary data obtained from the KHP survey that is publicly available on the survey website (https://www.khp.re.kr:444/eng/main.do). All the interviewees in the survey were anonymous, and all procedures involving human participants followed the ethical standards of the relevant institutional and National Research Committee and of the 1964 Declaration of Helsinki and its later amendments or comparable ethical standards. Ethical review and approval was not required for the study.

## Author Contributions

WC conceived and completed the study and accessed and verified the data.

## Conflict of Interest

The author declares that the research was conducted in the absence of any commercial or financial relationships that could be construed as a potential conflict of interest.

## Publisher's Note

All claims expressed in this article are solely those of the authors and do not necessarily represent those of their affiliated organizations, or those of the publisher, the editors and the reviewers. Any product that may be evaluated in this article, or claim that may be made by its manufacturer, is not guaranteed or endorsed by the publisher.
